# The Hedgehog Signaling Pathway: Where Did It Come From?

**DOI:** 10.1371/journal.pbio.1000146

**Published:** 2009-06-30

**Authors:** George Hausmann, Christian von Mering, Konrad Basler

**Affiliations:** 1 Institute of Molecular Biology, Universität Zürich, Zürich, Switzerland; 2 Swiss Institute of Bioinformatics, Universität Zürich, Zurich, Switzerland; Stanford University, United States of America

## Abstract

The Hedgehog signaling pathway plays a crucial role in development and disease. Its putative origins in an ancient system involved in regulating bacterial lipid transport and homeostasis offers clues about how the pathway might work today.

Complex body plans require sophisticated cell–cell signaling pathways. How these pathways evolved is often not very well understood. Here, we argue that the Hedgehog (Hh) signaling pathway may have arisen from systems that were originally designed for the transport and homeostasis of certain bacterial sterol analogs—the hopanoids. We propose a possible scenario for the evolution of Hh signaling and discuss how evolutionary considerations can shed light on the mysterious communication between the membrane-bound Hh transducers Patched (Ptc) and Smoothened (Smo).

The importance of the Hh signaling pathway has long been documented, even in Greek mythology; the tale of the one-eyed Cyclops was likely inspired by rare birth defects related to reduced Hh signaling [1],[2]. Whereas reduced Hh signaling can cause these and other developmental defects, inappropriate activation of Hh signaling contributes to certain forms of cancer, including basal cell carcinoma, the most commonly occurring form of skin cancer [3]. Both effects reflect the essential role of Hh in the control of patterning and growth during development and in the adult animal (for more detailed reviews, see [4]–[6]).

The current model for the production, transport, and transmission of the Hh signal is summarized in Figure 1. Two important components acting at the cell membrane are Ptc, which is the likely receptor for Hh, and the seven-pass transmembrane protein Smo, which acts downstream of Ptc. In the absence of Hh, Ptc blocks Smo activity. How this repression is achieved, and subsequently overcome by the Hh ligand, remains a mystery. One key to solving it may lie in understanding the multiple connections of the Hh pathway to lipid metabolism (reviewed in [7]). In addition, we argue that evolutionary considerations can identify a possible scenario for the origin of Hh signaling. To begin addressing both aspects, we start with a detailed look at Ptc and Smo.

**Figure 1:**
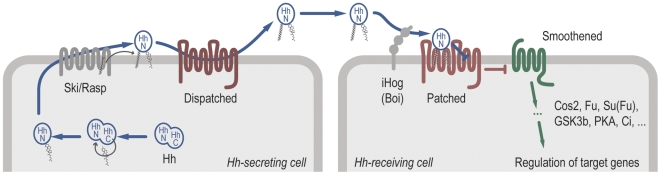
Schematic overview of the Hedgehog signaling pathway. Signal-secreting cells (left) release the morphogen protein Hh after modifying it through the addition of two lipid molecules. A C-terminal cholesterol moiety is added via the activity of an intein domain within Hh itself, whereas the protein Ski/Rasp attaches an N-terminal palmitic acid. Lipid-modified Hh is released from the producing cell with the aid of the Disp protein. Signal-receiving cells (right) bind Hh via the transmembrane protein Ptc, perhaps with the assistance of the iHog/Boi family of proteins. Hh binding to Ptc leads to the de-repression of the GPCR-related protein Smo. Smo subsequently initiates intracellular signal transduction events, which involve proteins such as Cos2, Fu, and Su(fu), that lead to changes in target gene expression. The inhibition of Smo by Ptc is of particular interest here; it occurs nonstoichiometrically, in a manner that appears to rely on a catalytic activity in Ptc.

## Evidence for Ptc-Like Proteins in Bacteria

**Figure 2:**
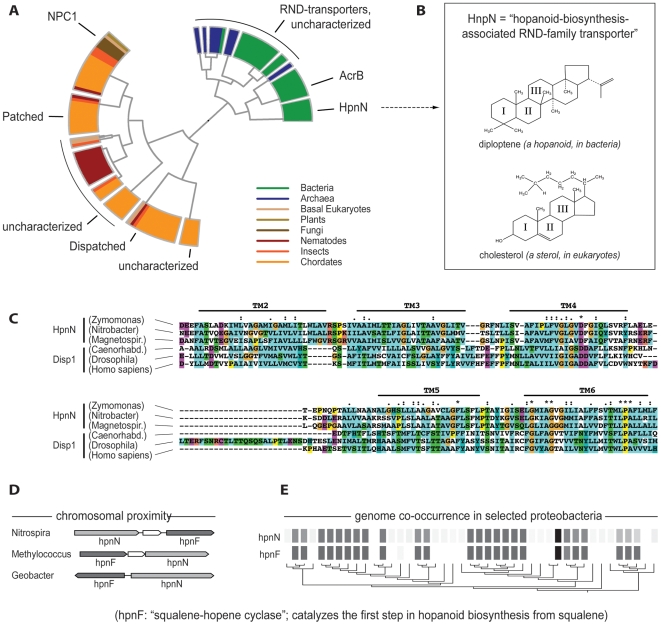
Ptc and Disp, two key proteins the Hh pathway, in their evolutionary context. (A) A phylogenetic tree of proteins related to Patched, limited to proteins that are full-length (i.e., those that contain all 12 transmembrane segments), is shown. The tree is color-coded according to the taxonomy of the organisms in which the respective proteins are found. Note one particular family of deeply branching bacterial Ptc homologs, the HpnN family, which encodes a transporter that is predicted to be associated with hopanoid biosynthesis. (B) A typical hopanoid is shown, next to cholesterol, a typical sterol. (C) Sequence alignment of selected HpnN family members with the most common reciprocal-best-match, the Disp family, is shown. Only six sequences are shown (three bacterial and three eukaryotic proteins); the alignment is restricted to transmembrane segments 2 to 6, which form the so-called SSD. (D) and (E) Evidence is shown for a functional association between HpnN-family transporters and HpnF; the latter being the enzyme that catalyzes the first step of hopanoid biosynthesis. Both genes tend to be either present or absent together in a given genome in proteobacteria and occasionally occur in direct chromosomal proximity.

Ptc proteins are members of a large superfamily that includes bacterial and archaeal resistance-nodulation division (RND) transporters [8] (Figure 2). RND transporters are proton antiporters that catalyze the active transmembrane efflux of numerous substrates from the cell ([9]; proton antiporters, or “counter-transporters,” use the physiological proton gradient at the membrane to pump out their substrates in exchange for protons that are allowed to flow inside). Ptc-family proteins have 12 transmembrane segments and have been described as hybrids of an RND-derived domain and a second domain, the so-called “sterol-sensing domain” (SSD) [10],[11]. However, our re-analysis of this family suggests that this classification of two distinct protein domains is somewhat arbitrary. Most eukaryotic and prokaryotic members of the family can be aligned over their entire length (Figures S1 and S2), with two well-conserved central blocks—of five transmembrane regions each—clearly visible. While these blocks roughly correspond to the suggested SSD and RND domains, we propose that they most likely stem from an ancient, internal duplication within the gene (this is also evident from the internal symmetry in the three-dimensional RND protein structure [12]). Some eukaryotic proteins in the superfamily have lost one of these two halves (the C-terminal half), and the remaining N-terminal half has been shown to have a role in sensing sterols [10]. For this reason, the first half of the protein can indeed be defined as a SSD, but it is as much of RND origin as is the second half. Other full-length members of the Ptc superfamily in eukaryotes (Figure 2) include Niemann-Pick C1 protein (NPC1) and Dispatched (Disp) [10]. The former is thought to be involved in cholesterol homeostasis, whereas the latter acts in Hh signaling to facilitate the release of the cholesterol-modified Hh protein from Hh-secreting cells (Figure 1). Taken together, a parsimonious interpretation of the available sequence data is that all current Ptc-family proteins are indeed of ancient origin over their entire length, and that they represent the oldest traceable components of Hh signaling.

Might eukaryotic Ptc-family members still act as proton antiporters today (we think so) and, in that case, what is their substrate? In bacteria, the actual substrates of RND transporters are often not known, and they are generally assumed to be quite diverse. However, one particular RND subfamily, having unusually high sequence similarity to eukaryotic Ptc-family proteins, has been tentatively linked to an intriguing substrate: hopanoids (Figure 2A–2C).

Hopanoids are the structural and functional analogs of sterols and, like sterols, are synthesized from squalene precursors. Accordingly, members of this particular subfamily of RND transporters have been termed “hopanoid biosynthesis-associated RND transporters,” or “hpnN” [13]. Further strengthening this link is the observation that hpnN genes can sometimes be found immediately adjacent to hopanoid biosynthesis genes, presumably forming co-transcribed transcription units (operons; Figure 2D and 2E). Remarkably, most of these hpnN-type RND genes have a reciprocal–best-match relationship with Ptc-family proteins in eukaryotes (Figure S3), i.e., they show up with the highest sequence similarity in homology searches extending across the eukaryote/prokaryote division. This makes the hpnN-type transporters the best candidates for functional counterparts of Ptc-like proteins outside eukaryotes. While this observation of course does not establish any current or past substrate(s) for the eukaryotic proteins, it does imply that structural analogs of sterols can be substrates. From this, a prediction might be that transporting/sensing such molecules (or rather, cyclic terpenoids in general) might still be the conserved function of eukaryotic Ptc-family proteins.

## The Origins of Smo

In contrast to Ptc, Smo-like proteins cannot be traced beyond the eukaryotes. Smo is a member of the eukaryote-specific superfamily G-protein–coupled receptors (GPCRs) [14]; ligands for this superfamily are typically small molecules. Given that Ptc might act by transporting/extruding a lipophilic molecule, could Smo's ligand be a lipophilic molecule as well? The majority of described GPCRs do not have strongly lipophilic ligands, but some examples exist—for example, the opsin/retinal pair in the eye [15] and the leukotriene receptors [16]. Of note with respect to Hh signaling is that some GPCRs can even sense steroids, for example, the putative estradiol receptor GPR30 [17],[18].

As Smo seems to be a much more recent innovation than Ptc, what conceivable chain of events might have connected Smo to Ptc activity and led to Hh signaling as it appears today? Below, we present a possible, minimal evolutionary path that could have generated today's setup, including an explanation for the observed unusual double-inhibition in the pathway, in which Hh acts by inhibiting Ptc, and Ptc in turn functions by inhibiting Smo.

## A Possible Evolutionary Scenario

**Figure 3:**
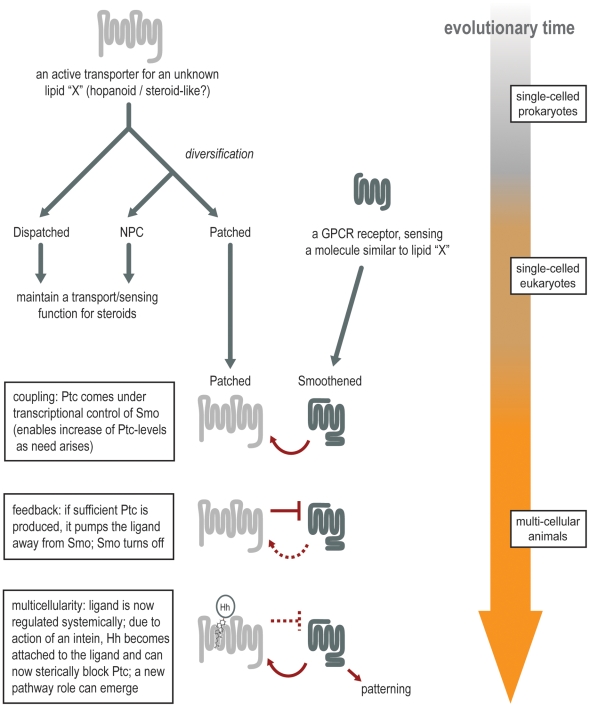
A parsimonious scenario for the evolution of the Ptc/Smo system. We hypothesize that during the transition to multicellularity, a pre-existing lipid homeostasis system took on a new function in signaling. Initially, an ancient lipid transporter diversified; one of its descendents came under the transcriptional control of a GPCR that sensed the same lipid (i.e., forming a negative homeostatic feedback loop). Then, the fortuitous addition of a protein moiety to the lipid in question brought the system under the control of gene expression; a neighboring cell could now secrete the lipid at will (by coupling it to the protein moiety). Because the combined lipid–protein molecule would block the transporter, this meant that the sending cell was capable of changing the perceived homeostatic state of the receiving cell, which would have established a graded (quantitative) mode of cell–cell communication.

We assume that the original function of Ptc was simply to transport an unwanted lipid molecule out of the cell. Smo, on the other hand, derives from a protein family whose main function is to sense and to transduce extracellular signals (i.e., the GPCR family). Therefore, we propose the following scenario: let us imagine that, in primitive eukaryotes, Smo was initially a receptor sensing lipid molecules and was acting upstream of the primitive Ptc transporter (Figure 3). The two molecules would have formed a simple homeostasis system; Smo would sense the abundance of a certain lipid and would transcriptionally induce Ptc whenever this lipid was in excess and needed to be removed from the membrane (i.e., pumped away). We propose that when multicellular organisms arose, this system was available and was recruited for a new purpose: cell-to-cell signaling.

There is evidence indicating that this repurposing could have been made possible via the fortuitous action of an intein. Inteins are a class of protein-coding genes that can insert themselves into other genes, and “splice” themselves out again after translation, joining the two fragments of the host protein together to restore its function. In the case of the Hh protein (whose C-terminal half encodes such an intein [19]), let us suppose that one of the two fragments to be joined happened to be a lipid moiety instead of a protein. The result would have been a lipid with a nonspecific, bulky protein domain (in this case, the future Hh N-terminal domain referred to as HhN) attached to it, and while that lipid would still have been a substrate for Ptc, the protein domain might now have blocked the transport mechanism, sterically, and prevented Ptc from pumping its normal substrate.

A consequence of this would have been that cells were now provided with a way to control the activity of the Ptc/Smo system in a neighboring cell. Simply by expressing and secreting Hh, they could sterically block Ptc in the neighboring cell. In other words, Ptc would sense and bind the cholesterol moiety of the secreted Hh (mistaking it for its ligand), but this binding would inhibit its exporter function due to the bulky protein attached to the cholesterol. This would in turn lead to the accumulation of the non-modified, normal ligand for Smo, because Ptc would no longer be available for depleting it. Importantly, this does not require any specific protein interface between Hh and Ptc (at least not initially). In the receiving cell, the only change that would have been necessary is the gradual addition of new target genes to the existing Smo pathway, in order to exploit this fortuitous new intercell “communication channel.” It should be noted that, from the outset, the new system would have been capable of graded (quantitative) responses; the original homeostasis system already had been capable of sensing different levels of the lipid, and the new ligand Hh would also be able to interfere to various extents, depending on the amounts produced and secreted.

## Predictions of This Evolving Model and How To Test Them

Our model is parsimonious, since only a few simple steps are required, and at each step a selectable advantage for the organism is conceivable. However, the model is also speculative—how can it be tested?

First, we propose that Ptc-family proteins are not only derived from RND transporters, but actually still work through the RND molecular mechanism. RND transporters are thought to function as trimers (three identical proteins are needed to form a functional unit in the membrane), and this arrangement is actually essential for the proposed molecular mechanism of pumping [20]. This is consistent with the proposition that catalytically active forms of Ptc are trimeric as well [21] and suggests they also require a proton gradient. Our model would predict that any prevention of trimerization should completely abolish repression of Smo by Ptc, while Hh binding might still be possible.

Second, we propose that at least some RNDs should be sterically inhibited by the covalent addition of bulky protein domains to their substrates; we think this is the case, because the substrate is generally thought to move “through” the RND and is extruded via an inner channel. This could be tested by adding bulky domains to RND substrates in bacteria or by replacing much of Hh with an unrelated but similarly sized protein domain which might still show (partial) activity in Hh signaling.

Third, we predict that the binding mode of Hh to Ptc was originally such that the cholesterol moiety would sterically fit into the original RND substrate pocket, and this might still be the case today. This could be tested by co-crystallizing an RND/hopanoid pair, as well as a Hh/Ptc pair, and comparing their binding modes. However, it is worth noting that, today, the cholesterol modification of Hh is no longer essential, so we assume that Ptc and Hh have evolved a specific protein interface in the meantime.

Fourth, our model makes predictions with respect to the surprising observation that *Caenorhabditis elegans*, while still having a clear ortholog of Ptc (Figure 2), lacks a homolog of Smo. As such, nematodes seem to represent an incomplete state of the pathway, and it might be tempting to try to use them to reconstitute aspects of signaling in organisms such as *Drosophila*. Would the *C. elegans* Ptc molecule, when introduced into *Drosophila*, show any effect on *Drosophila* Smo? From our model, we would predict that it might indeed be able to repress Smo (since we propose that it transports a ubiquitous lipid), but importantly, it should not specifically bind Hh—at least not when Hh is devoid of cholesterol.

Fifth, our model posits that Ptc works by transporting an agonist away from Smo (as opposed to transporting an “antagonist” towards Smo). This predicts that the action of Ptc on Smo is strictly cell-autonomous, and that Ptc and Smo need to co-localize for Ptc to function, at least in all those subcellular structures where Smo would otherwise be competent to signal.

Lastly, the communication between Ptc and Smo would happen via locally restricted lipid/sterol level fluctuations; we are essentially proposing that Ptc creates a local depletion of a specific membrane component. Perhaps this could be tested using lipidomics approaches on fractionated membrane samples, searching for changes in lipid abundances in response to Hh signaling or in response to Ptc overexpression. For this, interesting subcellular compartments would include lipid rafts, vertebrate cilia [22], or the late endosomes where Ptc and Smo co-localize [23]. It seems generally imperative that the location, movement, and site of action of Ptc and Smo are mapped with as much detail as possible, using the endogenous proteins at normal levels.

## Conclusions

The intriguing homology between components of lipid homeostasis pathways and components of the Hh signaling pathway leads to the hypothesis that the central membrane–players of the Hh signaling cascade—Smo and Ptc—evolved from a pre-existing lipid-sensing/homeostasis pathway. We propose a model of simple evolutionary steps, which posits that Ptc acts by pumping an activator of Smo, rather than an inhibitor. This scenario is compatible with most experimental data so far. The step-wise construction of pathways from older, pre-existing modules is turning out to be a general theme in developmental biology [24]. The simple evolutionary model we propose here may be a good starting point, but of course the evolution of the pathway could easily have been much less straightforward, taking leaps and bounds that we have not envisioned. To generally think more along evolutionary lines may nevertheless help to explain some of the more “exotic” findings—here and elsewhere in developmental biology.

## Supporting Information

Figure S1 **Phylogeny of Patched-family proteins and related proteins.** This is the detailed version of the Ptc tree; a simplified summary of this tree is shown and discussed in Figure 2. Tree lines are colored according to the taxonomic classification of the organism that encodes the protein. The protein accessions (as used in the STRING database, version 7.1) as well as the protein annotations are indicated. To construct the tree, all proteins annotated as containing the Pfam-domain Patched were extracted from STRING. Furthermore, homology searches with these proteins as queries yielded about 30% additional proteins (remote homologs). This search was conducted with the Smith-Waterman algorithm; homologs were maintained if they either had an alignment score above 100 bits or showed a reciprocal–best-hit to human Disp1. Sequences that were not full length, as well as a handful that showed unusually derived sequences (long branches), were removed manually. Sequences were aligned using Probcons, blocked using Gblocks and then used for tree reconstruction by PhyML. This figure can be magnified to improve readability.
(3.17 MB TIF)

Figure S2 **Multiple sequence alignment of eukaryotic Dispatched proteins and bacterial HpnN-type proteins.** The alignment shown here is a reduced version of the full alignment supporting the phylogenetic tree in Figure 2. Three representative Disp1 proteins and three representative HpnN-type were chosen and extracted from the full alignment. All aligned positions were maintained, but positions that showed gaps for all six sequences were removed. Putative transmembrane sections are shown, as predicted by PolyPhobius (PolyPhobius was run with the reduced alignment shown here as input).
(9.77 MB TIF)

Figure S3 **Close relatives of Patched/NPC1/Disp in Proteobacteria have a link to hopanoid biosynthesis.** Hopanoids are bacterial analogs of sterols—with a similar function in the membrane—and, like sterols, are synthesized from squalene. The most important enzyme for their biosynthesis is squalene-hopene cyclase (shc/hpnF), which catalyzes the first step of the biosynthesis pathway. In proteobacteria (out of which eukaryotic mitochondria originated), this enzyme gene co-occurs with a specific member of the RND superfamily. This co-occurrence pattern is complex (i.e., it is not dictated by the phylogenetic tree; even close relatives tend to vary, having either both genes or none). In addition, three independent instances of gene neighborhood can be observed (operons). Invariably, the partnered RND gene has a best–reciprocal-hit relation to eukaryotic members of the Patched/NPC1/Disp family in homology searches. Note that most of the bacterial genomes shown have several other RND-family genes besides hpnN, but these usually do not have such a best–reciprocal-hit relation to eukaryotes.
(1.91 MB TIF)

## Abbreviations

Disp: Dispatched
GPCR: G-protein–coupled receptor
Hh: Hedgehog
hpnN: hopanoid biosynthesis-associated RND transporters
Ptc: Patched
RND: resistance-nodulation division
Smo: Smoothened
SSD: sterol-sensing domain
