# Supplementary figures and images for: The Hedgehog Signaling Pathway: Where Did It Come From?
Source: PLoS Biol. 2009 Jun 30;7(6):e1000146. doi: 10.1371/journal.pbio.1000146 (PMC2698682; doi:10.1371/journal.pbio.1000146)

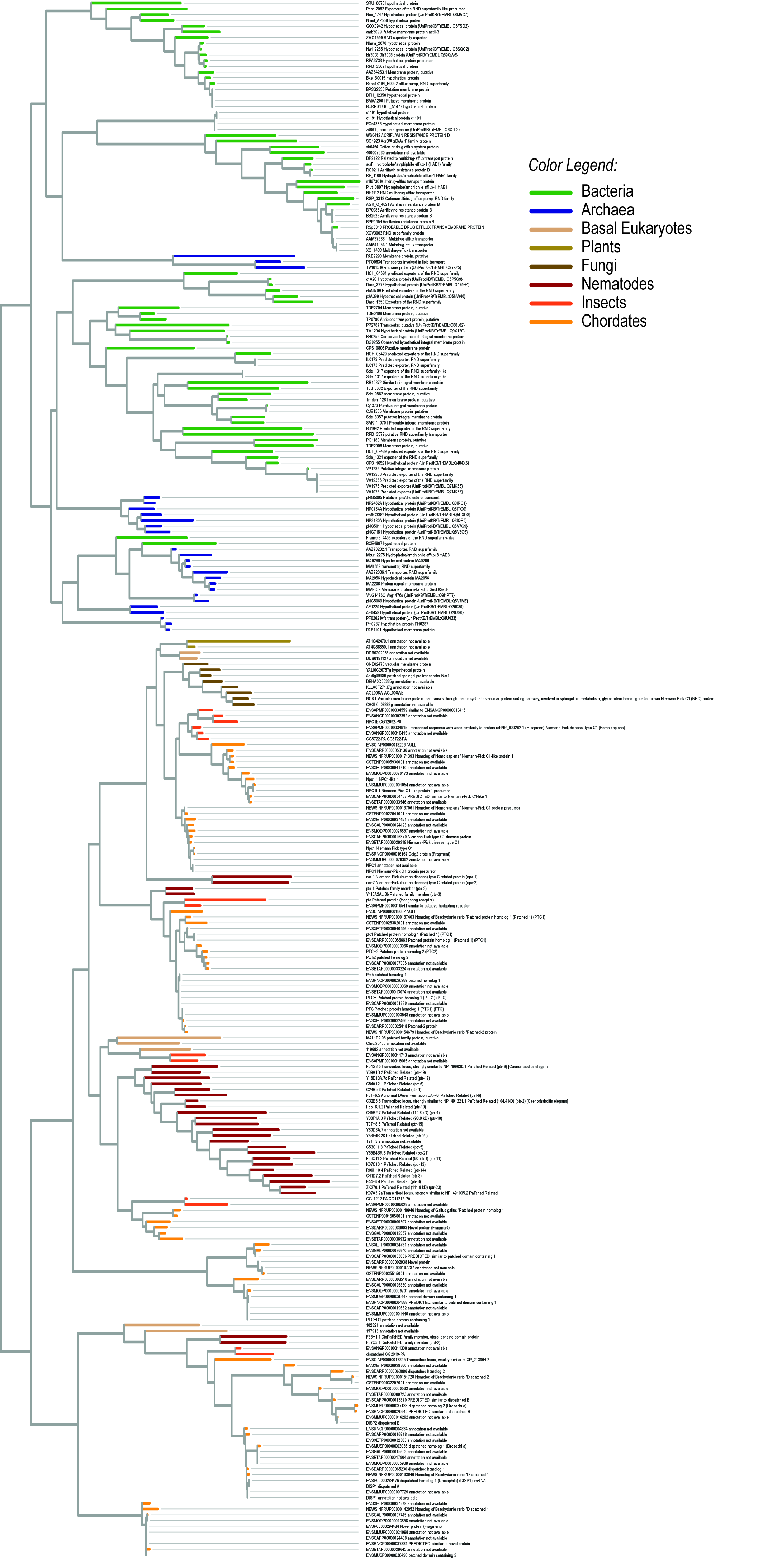

Supplement: Figure S1 — Phylogeny of Patched-family proteins and related proteins. This is the detailed version of the Ptc tree; a simplified summary of this tree is shown and discussed in Figure 2. Tree lines are colored according to the taxonomic classification of the organism that encodes the protein. The protein accessions (as used in the STRING database, version 7.1) as well as the protein annotations are indicated. To construct the tree, all proteins annotated as containing the Pfam-domain Patched were extracted from STRING. Furthermore, homology searches with these proteins as queries yielded about 30% additional proteins (remote homologs). This search was conducted with the Smith-Waterman algorithm; homologs were maintained if they either had an alignment score above 100 bits or showed a reciprocal–best-hit to human Disp1. Sequences that were not full length, as well as a handful that showed unusually derived sequences (long branches), were removed manually. Sequences were aligned using Probcons, blocked using Gblocks and then used for tree reconstruction by PhyML. This figure can be magnified to improve readability. (3.17 MB TIF) [file pbio.1000146.s001.tif]

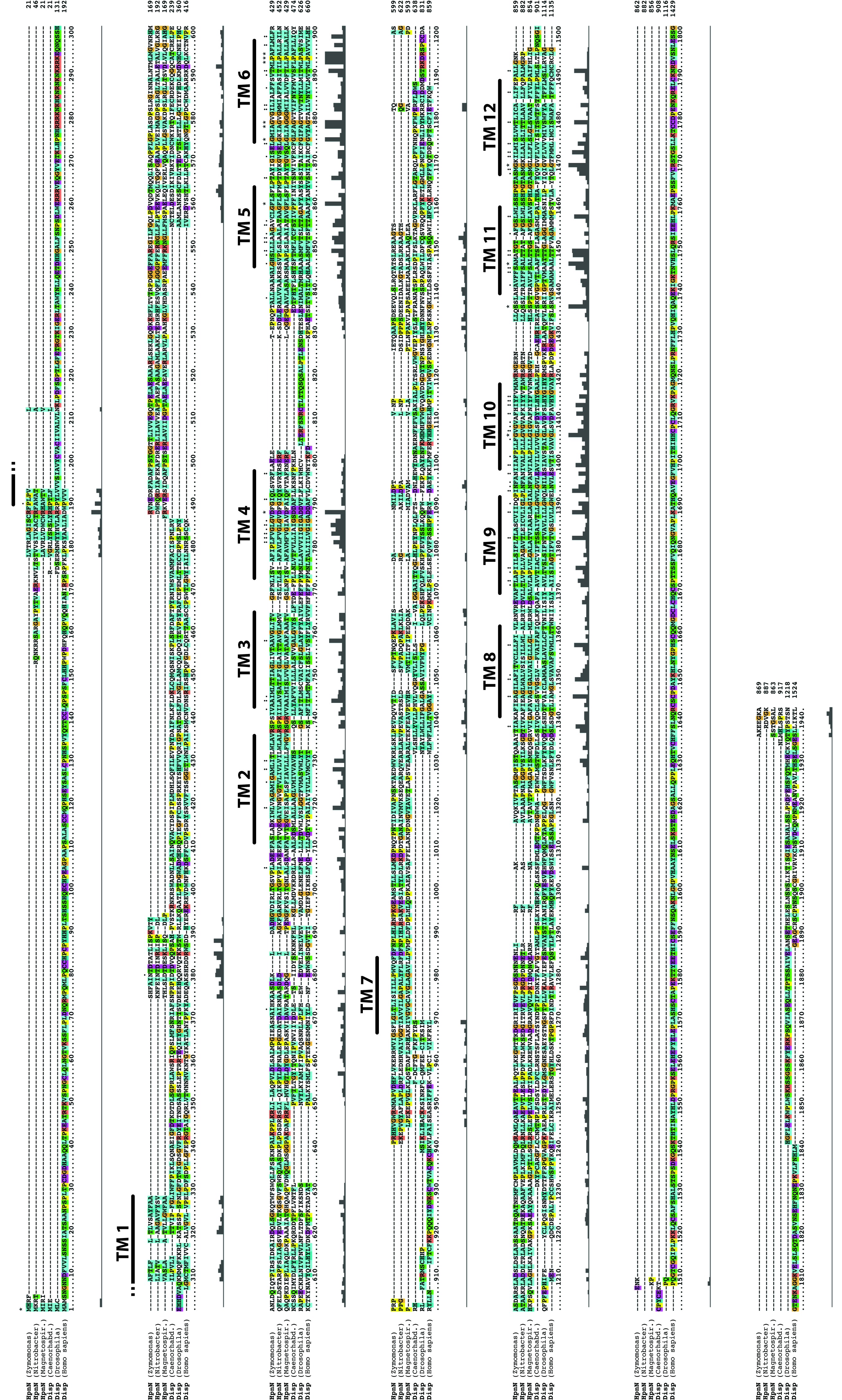

Supplement: Figure S2 — Multiple sequence alignment of eukaryotic Dispatched proteins and bacterial HpnN-type proteins. The alignment shown here is a reduced version of the full alignment supporting the phylogenetic tree in Figure 2. Three representative Disp1 proteins and three representative HpnN-type were chosen and extracted from the full alignment. All aligned positions were maintained, but positions that showed gaps for all six sequences were removed. Putative transmembrane sections are shown, as predicted by PolyPhobius (PolyPhobius was run with the reduced alignment shown here as input). (9.77 MB TIF) [file pbio.1000146.s002.tif]

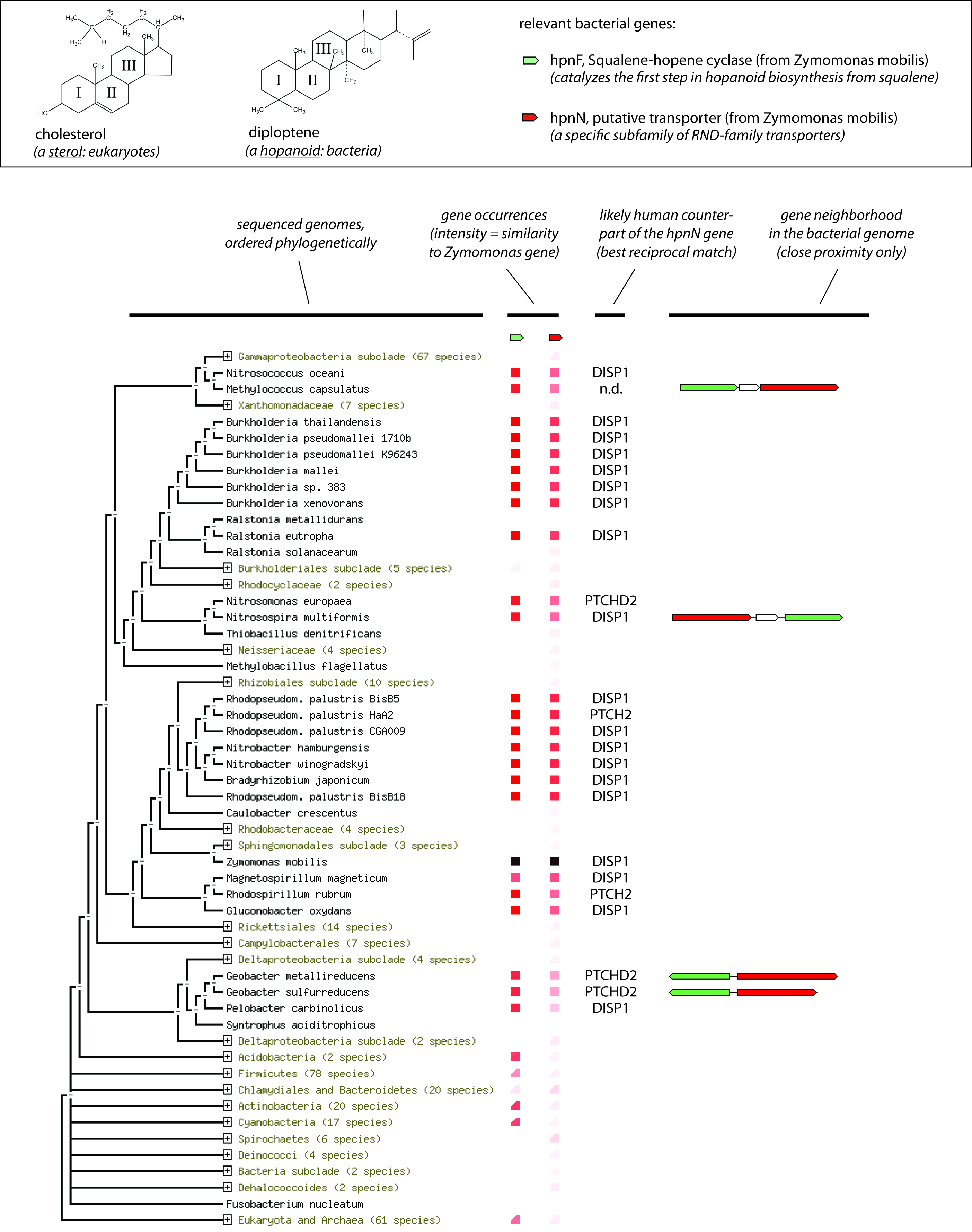

Supplement: Figure S3 — Close relatives of Patched/NPC1/Disp in Proteobacteria have a link to hopanoid biosynthesis. Hopanoids are bacterial analogs of sterols—with a similar function in the membrane—and, like sterols, are synthesized from squalene. The most important enzyme for their biosynthesis is squalene-hopene cyclase (shc/hpnF), which catalyzes the first step of the biosynthesis pathway. In proteobacteria (out of which eukaryotic mitochondria originated), this enzyme gene co-occurs with a specific member of the RND superfamily. This co-occurrence pattern is complex (i.e., it is not dictated by the phylogenetic tree; even close relatives tend to vary, having either both genes or none). In addition, three independent instances of gene neighborhood can be observed (operons). Invariably, the partnered RND gene has a best–reciprocal-hit relation to eukaryotic members of the Patched/NPC1/Disp family in homology searches. Note that most of the bacterial genomes shown have several other RND-family genes besides hpnN, but these usually do not have such a best–reciprocal-hit relation to eukaryotes. (1.91 MB TIF) [file pbio.1000146.s003.tif]
